# Efficacy and safety of portable ultrasound-guided pingyangmycin sclerotherapy for deep-seated oral and maxillofacial venous malformations: a retrospective observational study

**DOI:** 10.3389/fneur.2026.1932113

**Published:** 2026-10-06

**Authors:** Qizhang Wang, Xiye Sun, Chengzhi Zhao, Junda Wu, Haiyan Bai, Jiawei Zheng

**Affiliations:** 1 State Key Laboratory of Oral Diseases, National Center for Stomatology, National Clinical Research Center for Oral Diseases, Department of Oral and Maxillofacial Surgery, West China Hospital of Stomatology, Sichuan University, Chengdu, Sichuan, China; 2 Department of Microbiology and Immunology, The Peter Doherty Institute for Infection and Immunity, University of Melbourne, Melbourne, VIC, Australia; 3 Department of Oromaxillofacial Head and Neck Oncology, Shanghai Ninth People’s Hospital, College of Stomatology, Shanghai Jiao Tong University School of Medicine, Shanghai, China

**Keywords:** oral and maxillofacial region, pingyangmycin, sclerotherapy, ultrasound, venous malformation

## Abstract

**Objective:**

Intralesional sclerotherapy is the mainstay treatment for venous malformations (VMs). This study aimed to assess the efficacy and safety of ultrasound-guided pingyangmycin sclerotherapy for outpatients with deep-seated oral and maxillofacial VMs.

**Methods:**

This retrospective study included 49 outpatients with symptomatic deep-seated VMs in the oral and maxillofacial region who received portable ultrasound-guided pingyangmycin sclerotherapy between August 2023 and September 2025. The treatment response analysis after sclerotherapy included lesion volume reduction and symptom improvement. Ultrasound was used to calculate lesion volume. The assessment of global discomfort from the perspective of patients was calculated using the 0–10 numeric rating scale (NRS). The presence of complications was recorded during follow-up.

**Results:**

Among the 49 patients, the average number of sessions for each patient was 2 (range, 1–5). All patients experienced symptom relief after sclerotherapy. The median post-treatment NRS score was 0 (range, 0–1), compared with a median of 5 (range, 2–10) before sclerotherapy. Regarding lesion volume, twenty-one lesions (42.86%) demonstrated excellent response (≥90% reduction). Twenty-three lesions (46.94%) presented moderate response (≥50, <90% reduction). Five (10.2%) lesions showed mild reduction (<50% reduction). The mean lesion dimensions after treatments were 1.54 × 0.87 × 0.96 cm, with a corresponding calculated volume of 0.47 cm^3^, compared with average dimensions 3.27 × 1.69 × 1.85 cm (volume, 3.59 cm^3^) before sclerotherapy. A mean volume decrease of 82% was observed among all patients. Paired analysis showed significant improvement in both lesion volume and NRS score after the sclerotherapy. No severe complications occurred in this study.

**Conclusion:**

Portable ultrasound-guided pingyangmycin sclerotherapy is a feasible and well-tolerated outpatient-based procedure for patients with deep-seated oral and maxillofacial venous malformations.

## 1 Introduction

Venous malformations (VMs) are the most common slow-flow vascular malformations located in the oral and maxillofacial region. The pathogenesis of VMs has not been fully elucidated. VMs are believed to be congenital anomalies, and most lesions originate from endothelial cells with *TEK* or *PIK3CA* gene mutations (1). Clinically, skin or oral mucosa with superficial lesions often appear as bluish-purple, soft, and compressible masses, whereas deep-seated lesions are mostly located within the muscles or muscle space, with the skin and oral mucosa appearing normal at the early stage (2). Progressively growing lesions may affect the surrounding normal tissue and generate symptoms such as pain, bleeding, and swelling, or serious complications such as obstruction of vital structures, functional impairment, and esthetic disfigurement (3). Deep-seated oral and maxillofacial venous malformations present significant therapeutic challenges due to their inaccessibility and high recurrence rates following surgical resection. Intralesional sclerotherapy is the gold standard treatment for oral and maxillofacial VMs. Currently, bleomycin, pingyangmycin, polidocanol, and sodium tetradecyl sulfate are the most frequently used sclerosants (4). Pingyangmycin is an antitumor antibiotic as well as a most well-known sclerosant. Previous clinical studies have confirmed the effectiveness and safety of pingyangmycin for treating vascular malformations, such as VMs and lymphatic malformations (5–7).

Imaging examinations are critical for the diagnosis and treatment of deep-seated VMs. Imaging guidance can provide the necessary information regarding lesion distribution and the anatomical relationship with the surrounding tissues (8). Ultrasound, a less-costly imaging guidance tool, can optimize sclerotherapy outcomes for patients with slow-flow vascular malformations (9–11). With the development of ultrasound equipment, smaller and more portable devices, such as laptop ultrasound and wireless palm ultrasound, offer the opportunity to conduct sclerotherapy for outpatients. While such portability offers theoretical advantages, the specific efficacy and safety profile of portable- ultrasound-guided pingyangmycin sclerotherapy for deep-seated venous malformations remains unexplored. It is unknown whether the relatively lower image resolution and Doppler sensitivity of portable devices might compromise the precision of intralesional injection. Thus, studies specifically evaluating the operational feasibility and clinical outcomes of this portable approach are urgently needed.

Therefore, this retrospective study was designed to evaluate the efficacy and safety of this portable technique. We hypothesized that portable ultrasound-guided pingyangmycin sclerotherapy would be a feasible treatment for outpatients with oral and maxillofacial deep-seated venous malformations, achieving promising clinical responses with acceptable and self-limiting adverse effects. To analyze treatment outcomes, demographic data, clinical symptoms, lesion volume, treatment sessions, sclerosant dosage, and complications were collected before and after sclerotherapy.

## 2 Materials and methods

### 2.1 Patient selection

This was a retrospective observational study. The diagnosis of deep-seated sporadic VMs was confirmed by medical history, physical examinations, and imaging examinations based on ISSVA definition (12). Patients with deep-seated oral and maxillofacial type I and II VMs according to Puig’s classification who suffered from pain, swelling, and enlargement met the inclusion criteria to receive pingyangmycin sclerotherapy. After reviewing the electronic medical record system, 72 patients diagnosed with deep-seated oral and maxillofacial VMs who received portable ultrasound-guided pingyangmycin sclerotherapy in the Department of Oral and Maxillofacial Surgery, West China Hospital of Stomatology, Sichuan University, from August 2023 to September 2025 were consecutively identified. Seventeen patients were excluded due to incomplete medical records, or missing baseline clinical or ultrasound imaging. Of the 55 eligible patients, 49 had complete pre- and post-treatment data and were included in the final analysis. Six patients were excluded from the final analysis because they were lost to follow-up after the initial treatment session (Figure 1). The protocol was approved by the Institutional Ethics Committee of West China Hospital of Stomatology (WCHSIRB-D-2025-042-R1) This study was conducted in accordance with the ethical guidelines of the Helsinki Declaration. Consent for routine clinical treatment was obtained before treatment in accordance with institutional clinical practice. For participation in the present study, written informed consent was obtained directly from competent adult participants. For pediatric participants, written informed consent was obtained from their parents or legal guardians. Written consent for publication was obtained separately from the participants or their parents/legal guardians, as appropriate, for the publication of potentially identifiable clinical images.

**Figure 1:**
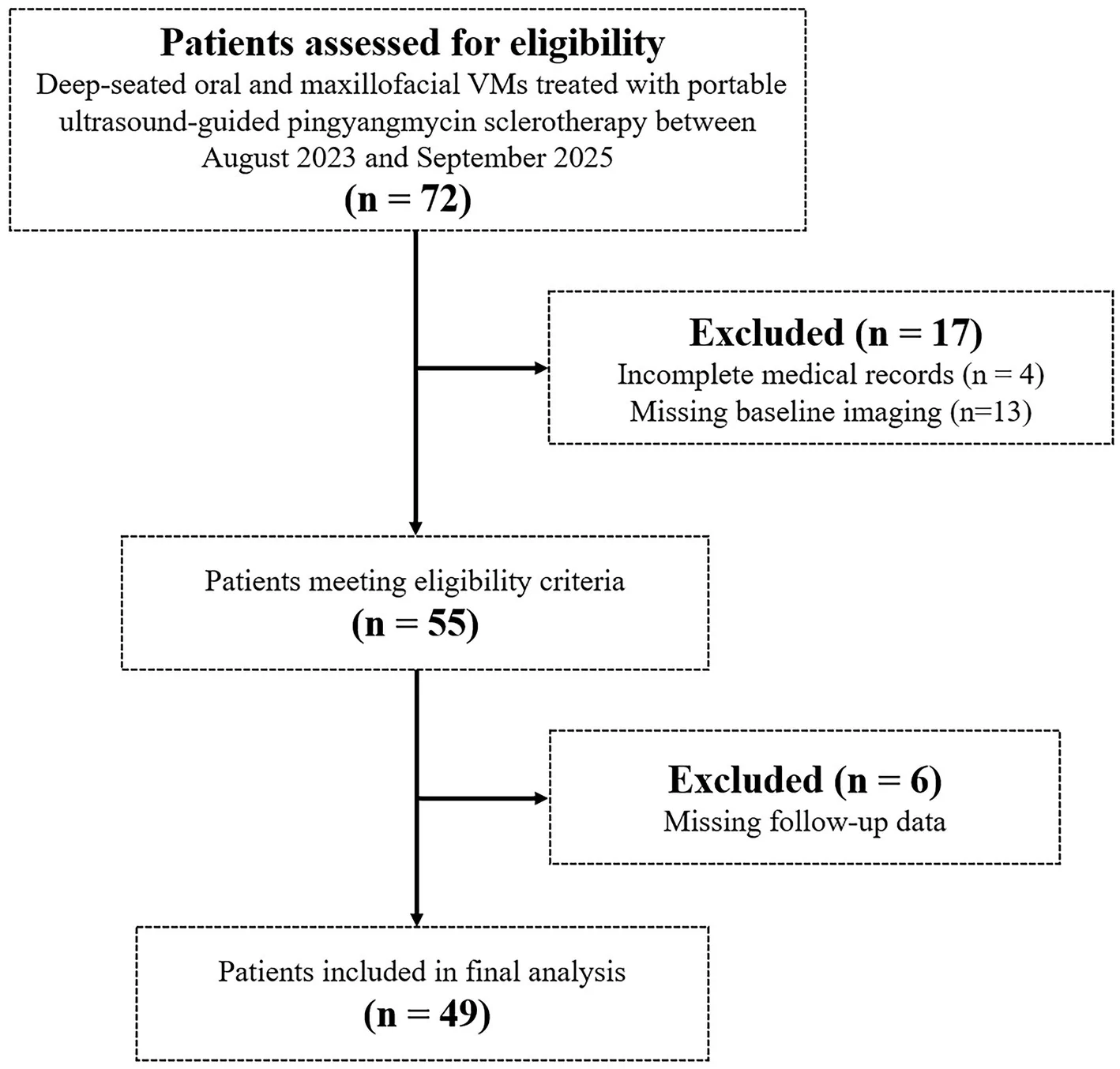
Patient selection flowchart.

### 2.2 Sclerotherapy procedure

All subjects underwent complete physical and imaging examination before pingyangmycin injection. All patients underwent color Doppler ultrasound examination at baseline and at each follow-up visit. For lesions that were equivocal on clinical examination or ultrasound, additional MRI was obtained for diagnostic confirmation at baseline. The pingyangmycin solution preparation is described below. Pingyangmycin powder (8 mg; Harbin Laiboten Pharmaceutical Co., Ltd., Harbin, China) was dissolved in 3 mL of 2% lidocaine, 4 mL saline, and 1 mL dexamethasone (5 mg) to reach a final concentration of 1 mg/mL (5, 13). The volume of pingyangmycin solutions per session depends on the lesion size and dose limitation. Ideally, the volume for a 1 cm^2^ lesion area ranges from 1 to 2 mL. The maximum pingyangmycin dosage per session was limited to 8 mg for adults and 4 mg for children (13, 14).

All procedures were performed in the outpatient treatment room under local anesthesia following the departmental standardized protocol for ultrasound-guided sclerotherapy. Before sclerotherapy, the puncture site and depth of transfusion needles were fully evaluated, and 2–6 skin or mucosa injection sites were labeled based on ultrasound images. After local sterilization, separate transfusion needles were inserted directly through skin or mucosa puncture sites under simultaneous ultrasound visualization (M9, Mindray, Shenzhen, China). A L12-4S linear transducer with a frequency range of 3–13 MHz was used. The penetration depth was adjusted dynamically between 1 and 3 cm. All Doppler examinations were performed using the **s**ystem’s pre-set superficial vascular protocol. As the needles gently advanced and reached the target dilated lumen, venous blood was pumped back into the needle and syringe. Subsequently, the pingyangmycin solution was slowly injected into the lesion, until the solution was uniformly distributed within the lesion (Figure 2). The amount of pingyangmycin solution injected was recorded. The injection sites were compressed locally, and patients were carefully monitored by clinicians for 30 min. All procedures were performed by the same senior surgeon using the same portable ultrasound device.

**Figure 2:**
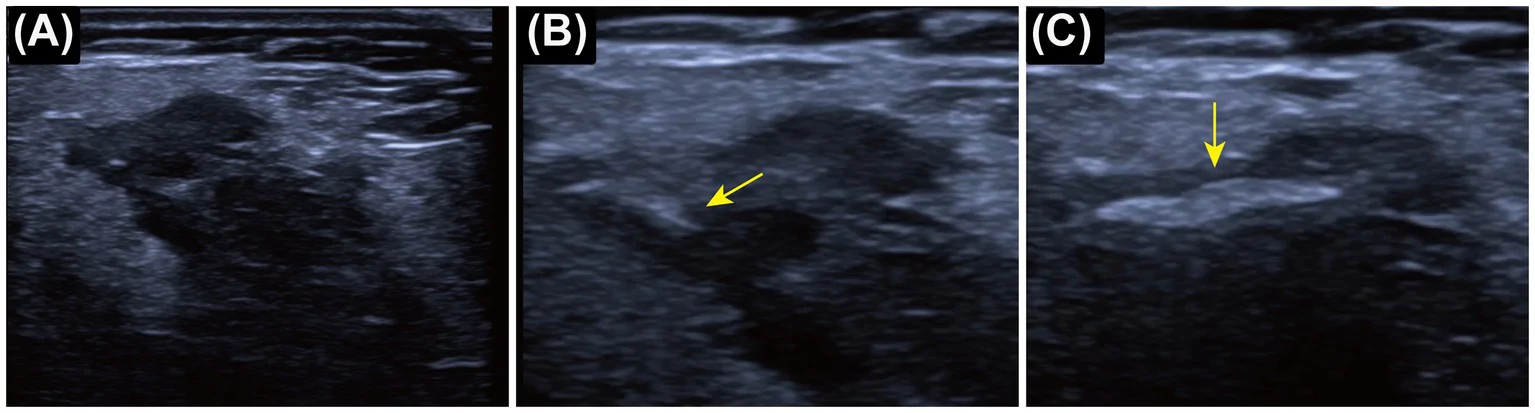
Sclerotherapy with pingyangmycin and portable ultrasound guidance. **(A)** Ultrasonography revealed the lesion location and depth before sclerotherapy. **(B)** Sclerotherapy with simultaneous ultrasound guidance demonstrated the insertion of a transfusion needle into the lesion. **(C)** Pingyangmycin was extended within the lesion.

All patients in this study received sclerotherapy at our outpatient clinic and were asked to undergo follow-up every 4 weeks. During the follow-up examinations, the need for extra sessions was fully assessed for each subject. Generally, personalized sclerotherapy consists of 1–5 sessions based on individual response and dose limitation. The total dosage of pingyangmycin should not exceed 40 mg for adults and 20 mg for children over 1 year. The end point was the disappearance of symptoms or lesions (6, 15). After the completion of sclerotherapy, patients were suggested to re-visit at an interval of 3 months.

### 2.3 Outcome analysis

The outcome analysis included demographic and clinical data, treatment response, and complications. Demographic and clinical data comprised gender, age, lesion location, treatment history, treatment session, and sclerosant dosage.

Regarding treatment response, lesion volume and clinical symptoms were considered crucial. Ultrasound was used to calculate lesion volume for each subject. Lesion dimensions in this study refer to the three orthogonal linear measurements reported in centimeters (cm). The mathematical equation for ellipsoid volume below were used to calculate the VMs lesion volume: V = π/6 × a × b × c (V: volume, a: the maximum diameter, b and c: the other two perpendicular diameters). Reduction rate = (preoperative volume - postoperative volume)/preoperative volume×100% (16, 17). The average reduction rate of all lesions was measured to represent the post-treatment mean volume decrease. The lesion volume reduction was classified as follows (10, 14): excellent (reduction of lesion volume ≥90%), moderate (reduction of lesion volume ≥50, <90%), mild (reduction of lesion volume <50%), and none (no discernible reduction of lesion volume). Three clinicians who were blinded to the pre- or post-treatment time point independently measured every lesion at both baseline and one month after the last treatment session. The final value for each dimension was derived as the arithmetic mean of the three independent measurements. The mean values were then used for volume calculation. Interobserver reliability was guaranteed by an agreement more than 80% for all variables assessed using the intraclass correlation coefficient.

Pre- and post-treatment clinical global discomfort involving pain, swelling, and any other bothersome sensations from the perspective of patients was measured using a 0–10 numeric rating scale (NRS), which is similar to the visual analog scale (2). Briefly, before the first treatment session, and one month after the last treatment session, each patient was given a printed form with the 0–10 scale, where 0 means ‘no discomfort at all’ and 10 means ‘the worst imaginable discomfort’. Patients marked their score independently without interference from clinical staff. The NRS scores were recorded by a research assistant who was not involved in the treatment procedures.

Adverse events were systematically assessed through medical record at each follow-up visit. Severe complications were defined as any adverse event requiring unplanned hospital admission, or resulting in permanent functional impairment or life-threatening conditions. Procedure-related symptoms, including self-limited pain, swelling, and mucosal ulceration, were recorded as expected adverse effects.

### 2.4 Statistical methods

Data collected for this study was analyzed using the SPSS software package (version 21.0; SPSS, Chicago, IL). The quantitative variables are presented as average, minimum, and maximum, or median, and interquartile range (IQR). The qualitative variables are presented as percentages. The normality of continuous variables (lesion reduction and NRS score) was assessed using the Shapiro–Wilk test. Since both variables deviated from a normal distribution (*p* < 0.05), paired comparisons were conducted using the Wilcoxon signed-rank test. Ordinal logistic regression analysis was conducted to detect any effects of relevant factors on treatment outcomes. The independent variables entered into the model were sex, age, and initial lesion size, while lesion reduction and treatment sessions were considered as dependent variables. Statistical significance was defined as *p* < 0.05.

## 3 Results

### 3.1 Characteristics of patients before sclerotherapy

A total of 49 patients were included in the final analysis. The average age of these 49 patients was 37.59 years, ranging from 10 to 73 years. The patient group consisted of 36 females and 13 males. Females accounted for 73.47%. All lesions occurred in the oral and maxillofacial regions: 14 (28.57%), 11 (22.45%), 18 (36.73%), 4 (8.16%), 1 (2.04%), and 1 (2.04%) lesion occurred in the parotid, submandibular, buccal, tongue, submental, and infraorbital regions, respectively. Thirteen patients had a history of previous treatment. Among these patients, ten underwent sclerotherapy, one received laser therapy, one received surgery, and one received both surgery and sclerotherapy. All these treatments were conducted >1 year before the current study (Table 1). All patients were symptomatic during their first visits to our hospital. Symptoms included discomfort, pain, body position-related enlargement, and facial asymmetry. The median pre-treatment NRS score was 5 (range, 2–10). They were fully evaluated and voluntarily decided to receive sclerosant injection. All lesion volume were measured before each sclerotherapy session. The average pre-treatment lesion dimensions were 3.27 × 1.69 × 1.85 cm, with a corresponding calculated volume of 3.59 cm^3^.

**Table 1:** Demographic data of patients.

Demographic data	No. (mean)	Percentage (range)
Gender		
Female	36	73.47%
Male	13	26.53%
Age (years)	37.59	10–73
Location		
Parotid	14	28.57%
Submandibular	11	22.45%
Buccal	18	36.73%
Tongue	4	8.16%
Submental	1	2.04%
Infraorbital	1	2.04%
Previous treatment		
None	36	73.47%
Sclerotherapy	10	20.41%
Laser therapy	1	2.04%
Surgery	1	2.04%
Sclerotherapy + Surgery	1	2.04%

### 3.2 Treatment efficacy and complications

Generally, 98 sclerotherapy sessions were conducted in 49 patients. The average session was 2 (range, 1–5). Sixteen (32.65%) patients had only one session, twenty-one (42.86%) patients had two sessions, nine (18.37%) patients had three sessions, two (4.08%) patients had four sessions, and 1(2.04%) patients had five sessions. Overall, they were injected with a total of 431.5 mg pingyangmycin. The mean dose for individuals was 8.81 mg (range, 2.5–24). An average of 4.4 mg pingyangmycin was used per session (range, 1.5–8 mg). Notably, all patients felt symptom relief after the treatments. The median post-treatment NRS score was 0 (range, 0–1). Forty-one patients (83.67%) were asymptomatic after sclerotherapy (NRS = 0), while 8 patients (16.33%) showed evident symptom improvement with only minor residual discomfort (NRS = 1). Regarding lesion volume, twenty-one lesions (42.86%) demonstrated an excellent response (Figure 3), with a reduction of more than 90%. Furthermore, twenty-three lesions (46.94%) presented a moderate response (Figures 4,5), whereas 5 (10.2%) lesions showed a mild reduction (Figure 6). The average lesion dimensions after the treatments were 1.54 × 0.87 × 0.96 cm, with a corresponding calculated volume of 0.47 cm^3^. A mean volume decrease (the average of individual percentage reductions) of 82% was observed among the 49 patients. The median follow-up period was 12 months (IQR, 4–15; range, 3–24 months) (Table 2). Follow-up data were available for all 49 patients at 3 months. Twenty-five patients (51.02%) completed at least 12 months of follow-up. Furthermore, paired comparisons concerning lesion reduction and NRS between pre-operative and post-operative values were subsequently conducted. Paired analysis showed significant improvement in both outcomes. The median lesion volume decreased significantly from 3.97 cm^3^ (IQR, 1.57–9.79) before treatment to 0.47 cm^3^ (IQR, 0.20–1.25) after treatment. The median of the paired differences (pre - post) was 3.02 cm^3^ (95% CI, 1.99 to 4.10; Z = 6.070; *p* < 0.001), corresponding to a large effect size (*r* = 0.867). The median NRS decreased significantly from 5 (IQR, 5–6) before treatment to 0 (IQR, 0–0) after treatment. The median of the paired differences (pre − post) was 5 (95% CI, 5 to 6; *Z* = 6.118; *p* < 0.001), corresponding to a large effect size (r = 0.874).

**Figure 3:**
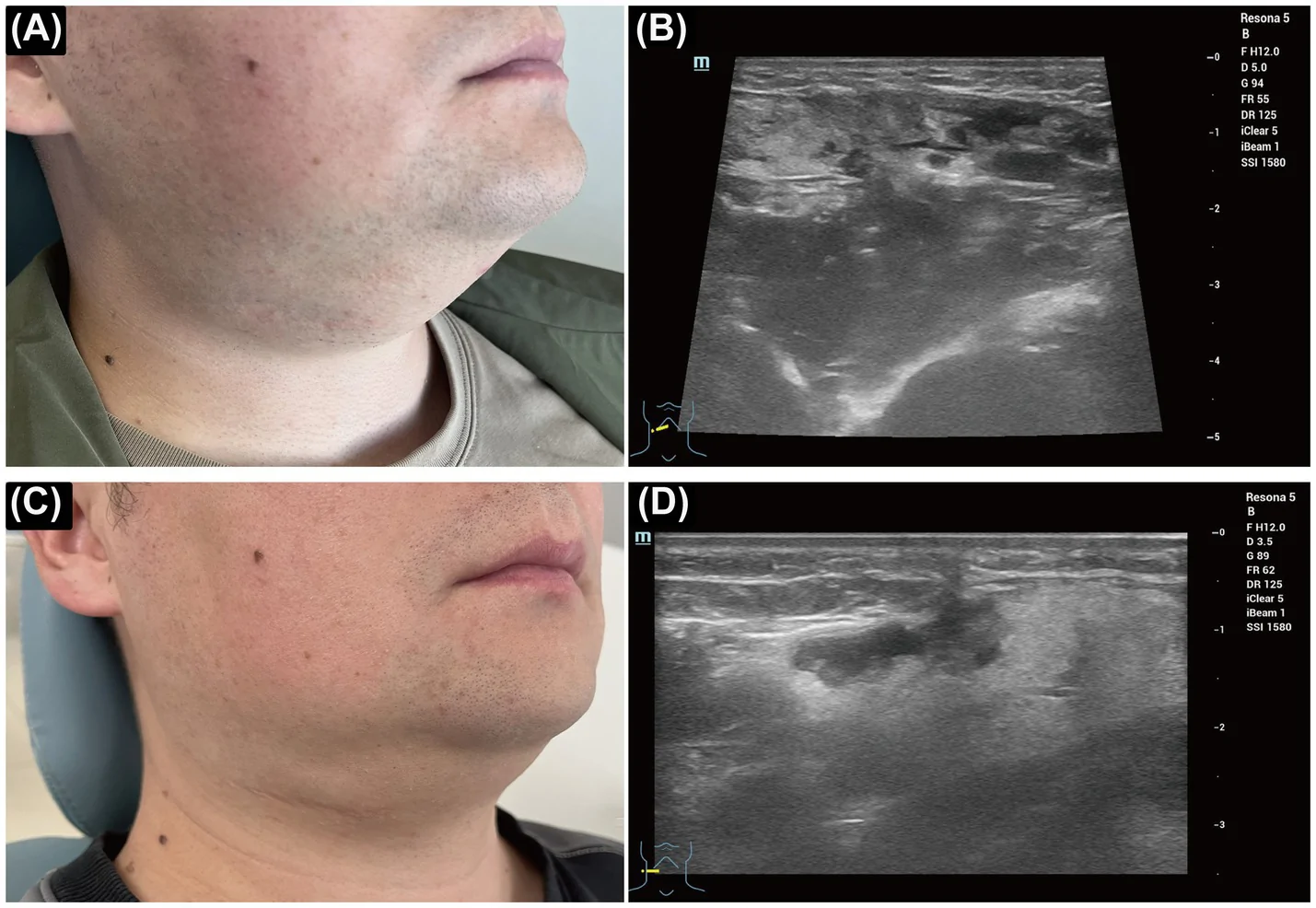
A 29-year-old male patient with right submandibular VM**. (A,B)** Clinical and ultrasound images before sclerotherapy. **(C,D)** Clinical and ultrasound images after 3 sessions of pingyangmycin sclerotherapy. Post-treatment NRS score = 0. Reduction of lesion volume = 97%.

**Figure 4:**
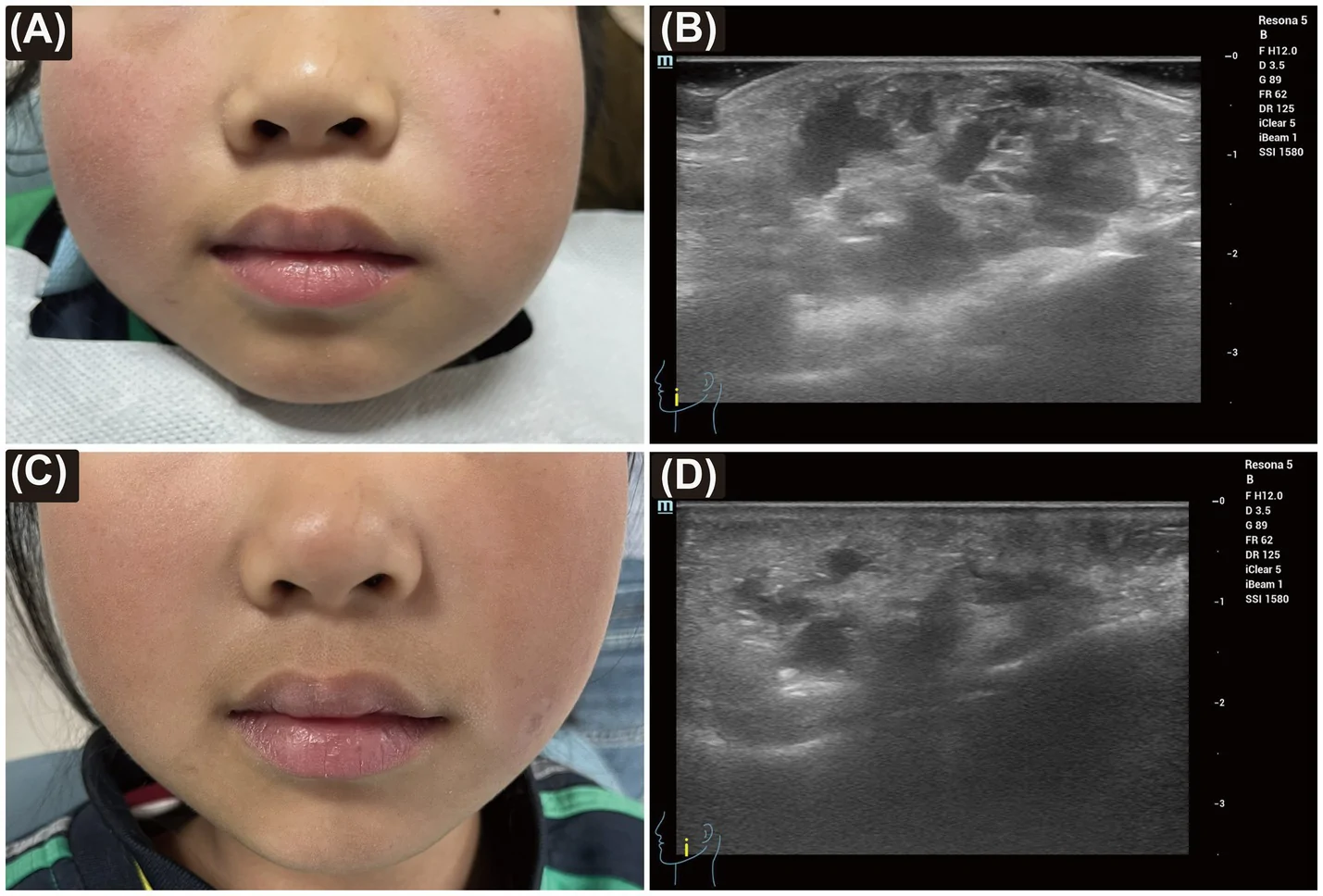
A 10-year-old female patient with left facial VM. **(A,B)** Clinical and ultrasound images before sclerotherapy. **(C,D)** Clinical and ultrasound images after 2 sessions of pingyangmycin sclerotherapy. Post-treatment NRS score = 0. Reduction of lesion volume = 88%.

**Figure 5:**
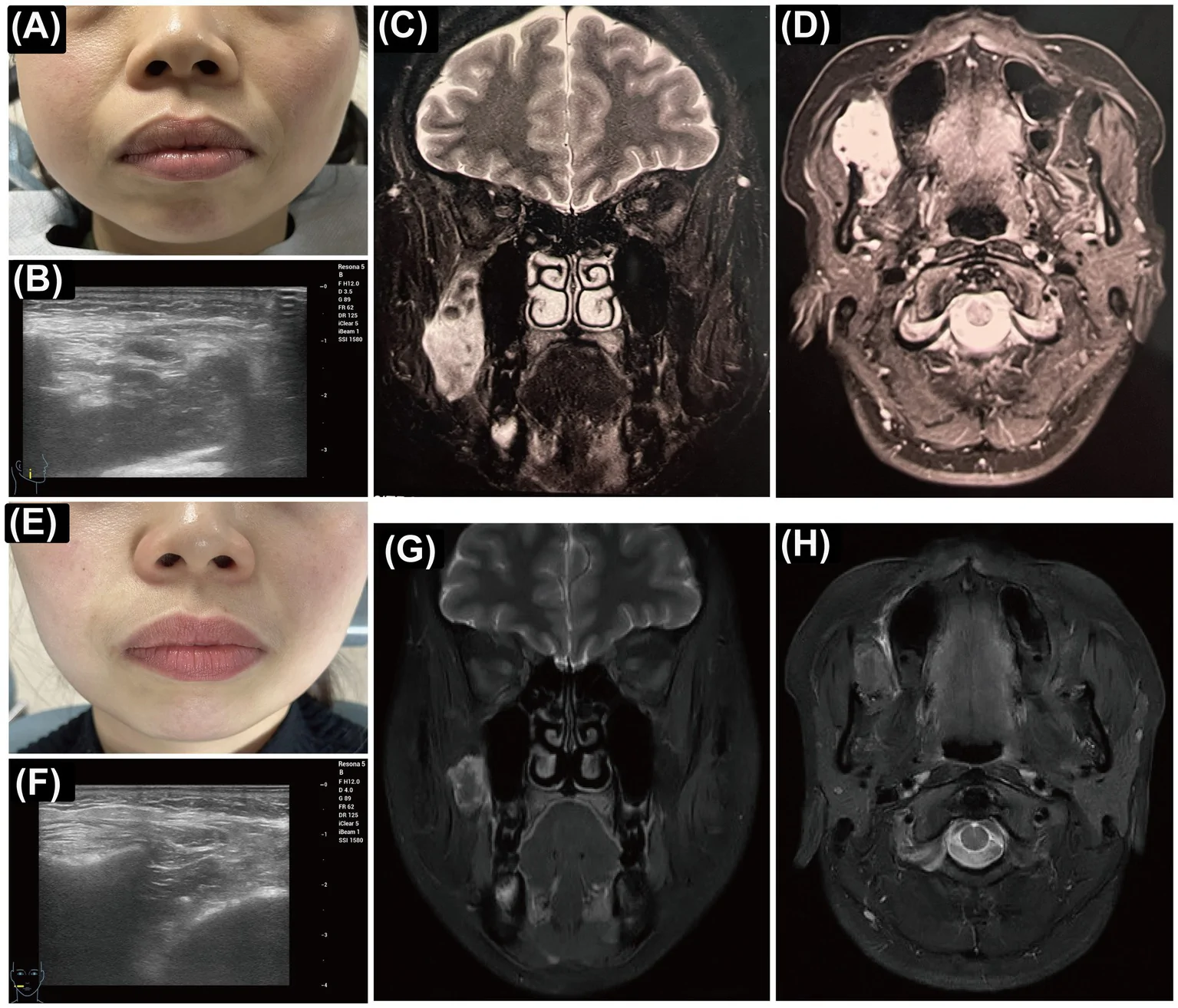
A 39-year-old female patient with right facial VM. **(A–D)** Clinical, ultrasound, and MRI images (coronal and horizontal plane) before sclerotherapy. **(E–H)** Clinical, ultrasound, and MRI images (coronal and horizontal plane) after 2 sessions of pingyangmycin sclerotherapy. Post-treatment NRS score = 0. Reduction of lesion volume = 78%.

**Figure 6:**
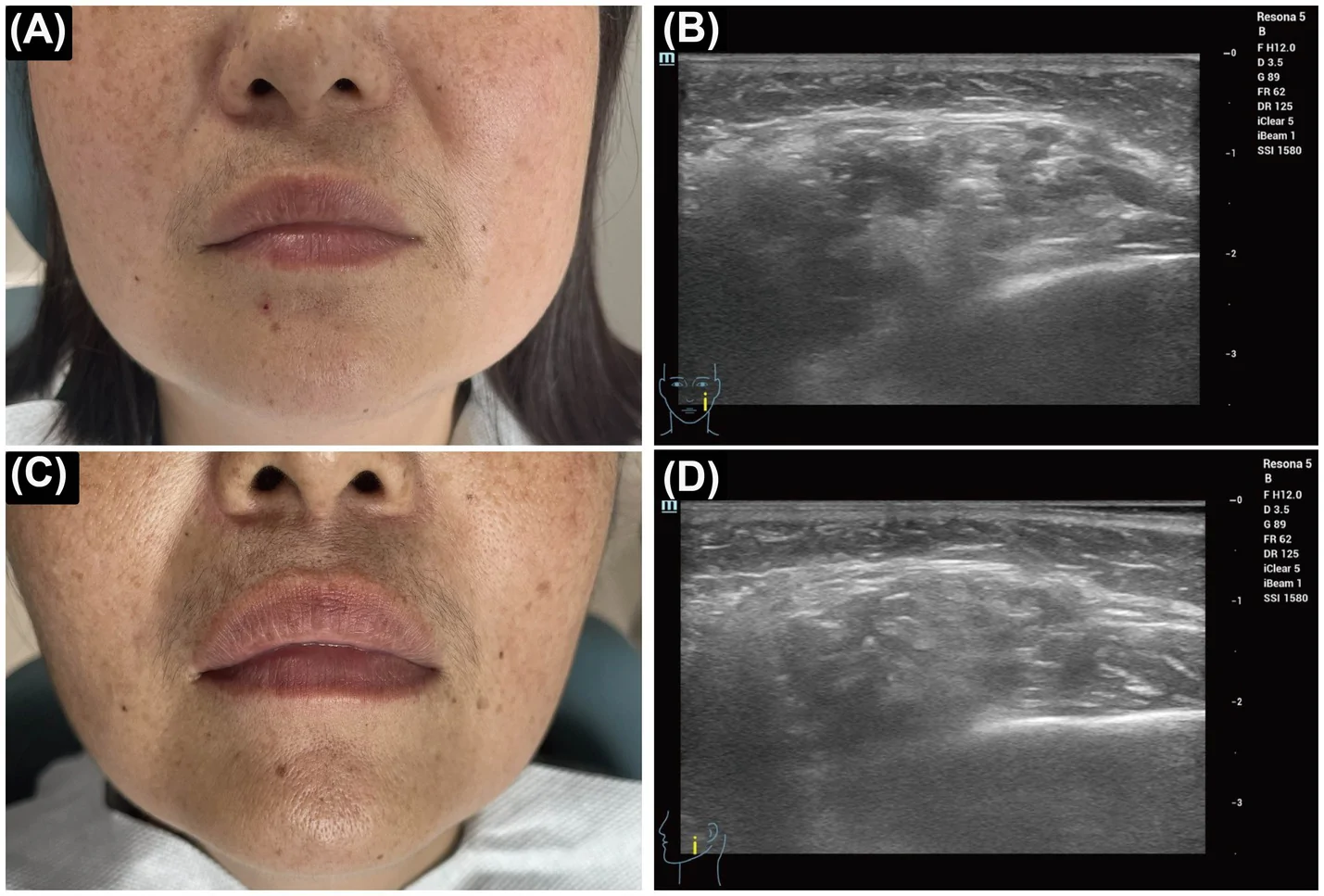
A 49-year-old female patient with left facial VM. **(A,B)** Clinical and ultrasound images before sclerotherapy. **(C,D)** Clinical and ultrasound images after 3 sessions of pingyangmycin sclerotherapy. Post-treatment NRS score = 0. Reduction of lesion volume = 47%.

**Table 2:** Clinical data of patients.

Clinical data	No. (mean/median)	Percentage (range)
Pre-treatment NRS	5	2–10
Pre-treatment lesion dimensions (cm)	3.27 × 1.69 × 1.85	
Pre-treatment lesion volume (cm^3^)	3.59	
Treatment sessions		
One session	16	32.65%
Two sessions	21	42.86%
Three sessions	9	18.37%
Four sessions	2	4.08%
Five sessions	1	2.04%
Individual sclerosant doses (mg)	8.81	2.5–24
Reduction of lesion volume		
Excellent	21	42.86%
Moderate	23	46.94%
Mild	5	10.2%
Post-treatment lesion dimensions (cm)	1.54 × 0.87 ×0.96	
Post-treatment lesion volume (cm^3^)	0.47	
Post-treatment NRS	0	0–1
Follow-up (months)	12	3–24

Considering the post-therapeutical complications, no severe side effects, such as cutaneous or oral mucosa necrosis, allergic reaction, or pulmonary embolism, occurred in this study. Procedure-related adverse effects were common but transient. All 49 patients (100%) underwent self-limited pain and swelling after 72 sessions (73.47%) of sclerotherapy, which resolved spontaneously within 1 to 2 weeks without specific intervention. Three patients (6.12%) endured localized oral mucosal ulceration after 4 sessions (4.08%) of pingyangmycin injection, which completely healed within 2 weeks. No patient experienced unplanned hospitalization or permanent functional impairment for any adverse event.

### 3.3 Factors affecting treatment outcomes

To assess the factors influencing treatment outcomes, age, gender, and initial lesion volume were allocated to evaluate the associations with lesion contraction, and treatment session. The results showed that lesion contraction, which was categorized into 3 subgroups in this study (excellent, moderate, and mild), was not significantly related to age, gender, and primary lesion volume (*P* > 0.05). Treatment session, which was categorized into 3 subgroups in this study (single session, two sessions, and multiple sessions), was positively associated with the primary lesion volume (*P* < 0.05), but was not significantly associated with age and gender (Table 3).

**Table 3:** Factors influencing treatment outcomes.

Outcome variables	Variables	OR	95% CI	*p* value	VIF	Parallel-lines test *P* value
Treatment session	Male vs. female	1.557	0.406–5.977	0.519	1.037	
	Age	1.032	0.996–1.069	0.079	1.028	>0.05
	Initial lesion volume	1.321	1.142–1.529	<0.001	1.065	
Lesion reduction	Male vs. female	2.763	0.740–10.326	0.131	1.037	
	Age	0.990	0.959–1.022	0.543	1.028	>0.05
	Initial lesion volume	0.975	0.899–1.057	0.542	1.065	

## 4 Discussion

Sclerotherapy is suitable for most oral and maxillofacial VMs. Pingyangmycin (bleomycin A5) was originally discovered in the Pingyang county of China (18). Studies have confirmed its therapeutic effects on VMs, lymphatic malformations, capillary malformations, and infantile hemangioma (5, 19–21). Published results presented that pingyangmycin had superior efficacy and safety profiles than other sclerosants (22, 23). Pingyangmycin is a derivative of bleomycin with a similar structure and chemical composition. Recent evidence suggests that the efficacy of pingyangmycin is comparable with that of bleomycin, but with fewer complications and lower cost (14). A meta-analysis involving 1,121 patients reported a minor complication rate of <10% for pingyangmycin, compared with approximately 26% for bleomycin (24), suggesting a more favorable safety profile for pingyangmycin. The occurrence of adverse effects was positively correlated with pingyangmycin concentration. Sclerotherapy with 2 mg/mL pingyangmycin resulted in more complications (25). Therefore, pingyangmycin (1 mg/mL) has become a common dosage for slow-flow vascular malformations in recent years. In this study, no severe side effects occurred after sclerotherapy with 1 mg/mL pingyangmycin. However, it should be noted that post-treatment local reactions were universal. All 49 patients underwent transient pain and swelling within 2 weeks, while 3 patients experienced self-limited oral mucosa ulceration. Collectively, the overall safety profile appears acceptable for outpatient settings.

According to previous studies, ultrasonography, computed tomography angiography (CTA), digital subtraction angiography (DSA), MRI, and fluoroscopy are all useful guiding devices (6, 8, 26, 27). In the present study, all patients underwent ultrasound pre- and post-treatment. Some lesions received additional MRI at baseline to confirm the diagnosis. After the sclerotherapy, MRI was not mandatory for patients because of several issues, such as higher cost, longer examination time for children. Accordingly, because ultrasound was the sole method used for pre-to-post treatment volume comparison in each patient, and the measurement protocol was strictly standardized, the calculation of lesion volume was conducted via ultrasound imaging. Imaging details including lesion depth, intramuscular or intermuscular location, and presentation of phleboliths, along with demographic and clinical data, and treatment response were summarized in (Supplementary Table S1).

The safety and efficacy of imaging-guided sclerotherapy for venous malformations have been well established in multiple series over the past decade, as summarized in (Supplementary Table S2). These previous studies, predominantly performed under general anesthesia with imaging guidance including DSA, conventional ultrasound, or fluoroscopy, have consistently reported favorable response rates and low complication profiles. In the present study, after an average of 2 sclerotherapy sessions, 89.8% of the patients showed more than half reduction of lesions, and the mean lesion volume decrease was 82%. Moreover, all patients experienced symptom relief after treatment. These findings are broadly consistent with previous reports. While the majority of published studies have employed fixed imaging systems under general anesthesia, we used a portable ultrasound device to guide injections in an outpatient clinic without sedation or general anesthesia. This approach avoids extra costs and risks related to general anesthesia and hospitalization, and shortens the treatment course. Adverse events in our cohort were generally self-limiting and acceptable, similar to previous reports. Overall, our findings indicate that portable ultrasound-guided pingyangmycin sclerotherapy appears to be a feasible and effective treatment option for outpatients. The observed clinical outcomes and safety profile in this study are broadly consistent with those reported in previous studies using conventional inpatient sclerotherapy. While our results suggest potential benefits in terms of convenience, accessibility, and healthcare resource utilization, formal validation through future comparative studies are required.

Sometimes it is ambiguous to determine when sclerotherapy should be discontinued. According to previous studies, no identical endpoint was found. In fact, many studies did not provide relevant information. Because deep-seated VMs in the oral and maxillofacial region are usually pervasive and lack a clear borderline with adjacent normal tissues, sclerotherapy cannot achieve complete reduction of lesions in most cases. The major symptoms of VMs are posture-related enlargement and pain, which always subside or entirely disappear when lesions shrink after sclerotherapy. Accordingly, the disappearance of symptoms is also strong evidence for the cessation of sclerotherapy. In this study, sclerotherapy accomplishment depended on two aspects: disappearance of either symptoms or lesions, which is in line with previous studies (6, 15). Regarding the symptom improvement, 83.67% patients were asymptomatic (NRS = 0) after sclerotherapy, while 16.33% patients showed only minor residual discomfort (NRS = 1). The median post-treatment NRS score was 0, which was profoundly improved in comparison with the median pre-treatment NRS score 5.

In this study, age, gender, and primary lesion volume were analyzed to determine whether they would affect treatment outcomes. Treatment session was positively related to the initial lesion volume, indicating that VM lesions with larger volume usually demand more sessions to acquire effective outcomes. Data showed that none of them were significantly correlated with lesion contraction. These results indicated that portable ultrasound-guided pingyangmycin sclerotherapy might be a relatively stable method for outpatients with oral and maxillofacial VMs. However, non-significant findings may reflect limited statistical power rather than true absence of association. Current evidence shows no sex discrepancy regarding the occurrence of VMs (28). However, it was intriguing to find that females accounted for 73.47% of the study population. Such an overwhelming ratio can be explained in different ways. First, the limited number of participants in this study cannot accurately reflect the actual sex composition. Moreover, oral and maxillofacial VMs usually cause facial enlargement and asymmetry. Females tend to care more about cosmetic issues and are willingly to receive therapy. Furthermore, VMs-related discomfort symptoms may be triggered by hormonal fluctuations (29), which are common in puberty, pregnancy, and menopause.

In this study, no recurrence was recorded during a median of 12 months of follow-up. Similarly, most relevant studies discovered no or rare recurrence within similar follow-up periods. Nevertheless, 13 of 49 patients in the present study had a history of sclerotherapy. All these procedures were completed more than 1 year before, and VMs relapsed before their visit to our department. This type of long-term recurrence is supported by other researchers. Anh et al. (30) reported that 3 (16.7%) patients with VMs recurred after polidocanol foam sclerotherapy in the following 1–4 years. Another study found a 20% recurrence rate after bleomycin sclerotherapy. In these 3 participants, none of them showed relapse within the 6-month follow-up. The recurrence all happened from 6 months to 2 years (24). For VMs treated with foam sodium tetradecyl sulfate, recurrence was observed in 12 (13.2%) patients during an average follow-up period of 23.5 months (31). Therefore, the limited follow-up period in the current study was insufficient to assess long-term recurrence and treatment durability. Even so, sclerotherapy is still recommended because relapse occurs less frequently than other intervention strategies. If regrowth happens, additional sclerotherapy sessions can be easily repeated at outpatient clinics without general anesthesia. Therefore, multiyear monitoring with sequential sclerotherapy sessions is suggested for patients with oral and maxillofacial VMs.

This study has several limitations. The small sample size could not accurately represent the actual treatment outcomes, and the limited follow-up time failed to capture any long-term recurrences or adverse effects. Furthermore, the consecutive enrollment strategy at a single institution may introduce selection bias. The inclusion of both pediatric and adult patients, patients with and without previous treatment history can affect the authenticity of results. Regarding the assessment of global discomfort, we acknowledge that the 0–10 numeric rating scale used in this study was adapted to measure global discomfort rather than a validated, pain-specific instrument. However, this adaptation has not undergone formal psychometric validation for the assessment of global discomfort. Moreover, the lack of comparisons constrained the comprehensive assessment of treatment efficacy and safety. While this case series demonstrates the outcomes of pingyangmycin therapy under portable ultrasound guidance in an outpatient setting, it does not establish superiority over alternative sclerosants, imaging guidance strategies, or treatment settings. Accordingly, our results should be viewed as descriptive observations in a selected cohort, and future controlled studies are needed to validate these preliminary findings.

This study was conducted at a single academic center with a dedicated interventional team, which may limit direct generalizability to other practice settings. Nevertheless, the use of portable ultrasound—a widely available, low-cost, and radiation-free imaging modality—suggests that our treatment protocol could be feasibly adopted in outpatient clinics, or resource-limited hospitals where DSA or MRI guidance is not readily accessible. Regarding age, this study included both children and adult patients, indicating that the protocol may be applicable across a broad age range. However, young children may require general anesthesia or sedation, so our findings should be extrapolated to this subgroup with caution. Importantly, our results apply specifically to venous malformations of oral and maxillofacial region. Whether this protocol is equally effective for lymphatic malformations, or mixed vascular anomalies remains unknown and requires separate investigation. Similarly, our results cannot be directly generalized to other sclerosants without further validation. Future multicenter studies with standardized protocols are warranted to confirm the external validity of our observations.

## 5 Conclusion

Portable ultrasound-guided pingyangmycin sclerotherapy was associated with favorable short- to mid-term clinical and volumetric outcomes in this selected outpatient cohort, with no severe complications observed. Controlled studies with standardized outcome assessment and longer follow-up are required to confirm comparative efficacy, safety, and long-term durability.

## Data Availability

The raw data supporting the conclusions of this article will be made available by the authors, without undue reservation.
